# Harnessing High Yield Potential in Wheat (*Triticum aestivum* L.) under Climate Change Scenario

**DOI:** 10.3390/plants12061271

**Published:** 2023-03-10

**Authors:** Hanif Khan, Harohalli Masthigowda Mamrutha, Chandra Nath Mishra, Gopalareddy Krishnappa, Ramadas Sendhil, Om Parkash, Arun Kumar Joshi, Ravish Chatrath, Bhudeva Singh Tyagi, Gyanendra Singh, Gyanendra Pratap Singh

**Affiliations:** 1 ICAR-Indian Institute of Wheat and Barley Research, Karnal 132001, India; 2 ICAR-Sugarcane Breeding Institute, Coimbatore 641007, India; 3 Department of Economics, Pondicherry University (A Central University), Puducherry 605014, India; 4 International Maize and Wheat Improvement Center (CIMMYT), New Delhi 110012, India; 5 Borlaug Institute for South Asia (BISA), New Delhi 110012, India; 6 ICAR-National Bureau of Plant Genetic Resources, New Delhi 110012, India

**Keywords:** wheat, yield potential, lodging, plant growth regulators, physiological traits

## Abstract

Wheat is a major staple food crop for food security in India and South Asia. The current rate (0.8–1.2%) of genetic gain in wheat is significantly shorter than the 2.4% needed to meet future demand. The changing climate and increased yield loss due to factors such as terminal heat stress necessitate the need for climate-resilient practices to sustain wheat production. At ICAR-Indian Institute of Wheat and Barley Research in Karnal, Haryana, India, a new High Yield Potential Trial (HYPT) was conceptualized and subsequently conducted at six locations in the highly productive North Western Plain Zone (NWPZ). An attempt was made to harness higher wheat yields through the best pipeline genotypes suitable for early sowing and modified agronomic practices to explore the feasibility of a new approach that is profitable to farmers. The modified agronomic practices included like early sowing, application of 150% recommended dose of fertilizers, and two sprays of growth regulators (Chlormaquate chloride and Tebuconazole) to prevent lodging. The mean yield in the HYPT was 19.4% superior compared to the best trials conducted during the normal sowing time. A highly positive and significant correlation of grain yield with grain filling duration (0.51), biomass (0.73), harvest index (0.75), normalized difference vegetation Index (0.27), chlorophyll content index (0.32), and 1000-grain weight (0.62) was observed. An increased return of USD 201.95/ha was realized in the HYPT when compared to normal sowing conditions. This study proves that new integrated practices have the potential to provide the best profitable yields in wheat in the context of climate change.

## 1. Introduction

Wheat, being a major staple food crop, plays an important role in food and nutritional security. It caters to ~20% of the calories and protein intake needs of humankind in most parts of the world, including South Asia. As such, there is a need for improving and sustaining yield potential in wheat to meet global food demand. Genetic improvements in yield continue in the world’s staple crops [1] but to realize the potential of these improvements in farmer’s fields under changing climate conditions requires improved agronomic practices [2]. Potential yield is defined as the yield of the best-adapted cultivar with the current best practices of agronomic management ensuring the absence of manageable abiotic and biotic stresses [3]. Potential yields in wheat are constrained in many climates by abiotic stresses, including terminal heat stress, water limitations, lodging, and salinity and biotic stresses such as rusts, foliar blight, aphid infestation, etc. In India, wheat loss due to lodging caused by un seasonal rains is reported to be in the range of 12–66% [4], while terminal heat stress in the last crop season (2021–2022) caused 3 MT wheat yield reduction in India [5]. Economic yield is the yield attained by farmers given the prevailing weather, but inputs and other crop management practices are applied at the economic optimum (maximizing margin); this may not necessarily coincide with the levels that produce a maximum yield, and this generally remains at approximately 75–85% of potential yield [6]. Modern plant breeding and advances in management practices have substantially contributed to the annual gain of around 0.8–1.2% in crop productivity [1,6]. Nevertheless, this rate of improvement is not sufficient to keep up with food and nutritional demands of the projected global population in 2050 [7] due to continuous challenges by climate change. By 2050, the projected yield reduction in India as a result of climate change ranges from 6 to 23% [8].

After the establishment of the All India Coordinated Wheat Improvement Project (AICWIP) in 1965, and with the support of programs pioneered by the N.E. Borlaug-led CIMMYT in Mexico, Indian wheat production and productivity made a breakthrough popularly known as the ‘Green Revolution’. Since then, major genes influencing adaptation, such as those controlling vernalization response, photoperiod sensitivity, plant height, and development rate, and “earliness per se” have been identified and incorporated into wheat varieties [9,10,11,12]. Overall, concerted efforts under India’s coordinated wheat project led to the development of widely adapted bread wheat varieties and have contributed significantly to India’s increased food supply year after year [13,14]. However, the yield potential of wheat genotypes in the breeding pipeline has not been properly assessed due to a fixed set of production practices. Also, breeding efforts in wheat were limited by insufficient knowledge about biology of key yield-contributing traits.

The full genetic potential of a genotype can be harnessed only under optimum production conditions. This is more necessary in regions like the North Western Plains Zone (NWPZ) of Indo-Gangetic plains; this area is around 12 million hectares and is one of the most productive regions of spring wheat cultivation in the world. In this region, which falls in the mega-environment1 (ME 1) [15], few efforts have been made to harness the potential yield of wheat varieties under early sowing, high fertility, non-lodging conditions through the application of modified crop management practices (growth regulations, etc.). The inferences drawn from this mega-production condition will have wider implications globally. Advancing the sowing period of a crop may be one of the most important climate-resilient strategies for yield optimization. Studies suggest that climate change has caused a shift in the optimum sowing window of wheat, pushing the window towards early sowing in the Indo-Gangetic states of India, where a delay in sowing result in yield reduction ranging from 36.09 to 70.80 kg ha^−1^ day^−1^ [16]. On the other hand, crop simulations reveal that early sowing combined with wheat varieties that mature slowly could exploit a longer growing season, thereby providing higher yields [17]. It has been proposed that early sowing systems can increase Australian wheat yields by 0.54 t ha^−1^ under climate change conditions [18]. Simple adaptation options, including a change in sowing times and increased doses and efficient use of fertilizer inputs, do not only offset yield reduction but could actually improve yields until the middle of the century [19]. The USAID and the Bill and Melinda Gates Foundation jointly established the Cereal Systems Initiative for South Asia (CSISA) in 2009 to help farmers to adapt to climate change and rainfall variability. This effort resulted in the success story of nearly 0.62 million farmers, who realized considerably higher yields due to their adoption of an early sowing mitigation strategy (https://www.usaid.gov/results-data/success-stories/saving-india%E2%80%99s-wheat-fields (accessed on 24 November 2022)). A recent study showed enhanced grain yield under early (October) sowing conditions in India [20].

Hence, in the present study, an effort was made to evaluate the maximum yield potential of wheat genotypes through a combination of practices, including new advanced genotypes with high genetic potential, early sowing, and appropriate agronomic practices, coupled with the use of growth regulators. Additionally, an attempt was made to determine the contributions of different factors for improved grain yield to judge cost-benefit analysis. This was performed so that an appropriate package of wheat production practices can be suggested to farmers for achieving higher yields under climatic challenges, especially terminal heat stress.

## 2. Results

### 2.1. Effect of Early Sowing and PGR on Grain Yield

**Figure 1:**
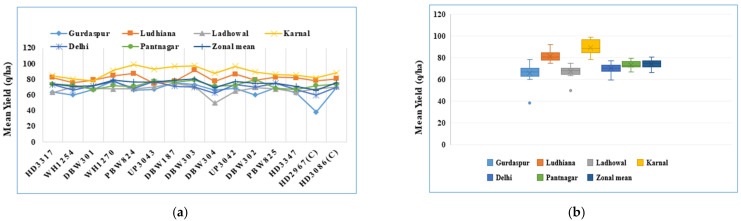
Graphical representation of mean yield of 15 varieties and 6 environments. (**a**) line graph illustrates varietal performance; (**b**) box plot illustrates environment-wise performance.

Multi-location data from the six centres were subjected to statistical analysis and are presented in Table 1. The highest trial mean yield of 89.0 q/ha was recorded at Karnal, while the lowest (65.7 q/ha) was recorded at the Gurdaspur location. DBW303 was the highest yielding (80.4 q/ha) genotype when compared to the zonal mean yield of 74.3 q/ha. DBW303 showed a 6.35% yield superiority over the best check, HD3086 (75.6 q/ha). DBW187 and WH1270 also showed significant yield superiority (3.97% and 3.84%, respectively) over the best check (HD3086), indicating their suitability and adaptability for early sowing and high fertility conditions. The other check genotype, HD2967, was found to be the lowest yielding (65.9 q/ha) (Table 1, Figure 1a,b). The zonal mean yield data (68.4 q/ha) from an agronomic trial conducted in the same locations for the same genotypes using the recommended dose of fertilizer (RDF) and the mean yield (62.2 q/ha) of an AVT-IR-TS trial in the same year were reported (Table 1) for yield comparison. The mean yield of the HYPT was significantly higher when compared to other trials.

### 2.2. Phenological, Physiological, and Yield Associated Traits

The data on phenological, physiological, and yield associated traits were recorded at the Karnal location to assess the traits that possibly contributed to higher yield in this trial (Table 2 and Table 3). Data analysis using the CD and Duncan test indicated significant variations among the traits as well as the genotypes. The values for the traits ranged from 103–121 days for DTH, 143–156 for DTM, 36–42 days for GFD, 0.76–0.82 for F_V_/F_M_, 25.3–41.2 for NBI, 1.0–1.58 for FLAV, 32–41.0 for CCI, 26.3–28.8 °C for CT, 0.882–0.9 for NDVI, 30.6–34.2 kg/plot for BM, 35.8–42.9 for HI, 42–65 for GNS, 2.0–3.2 for GWS(g), 40–62 for TGW(g), 95.7–109.5 cm for PH, 9.7–12.6 cm for SPKL, and 89–129 for TN; early sowing showed a significant positive impact on grain filling duration (GFD). The longest GFD (44.5 days) was observed for PBW824, followed by HD3717 (42 days), and DBW303, WH1254, and HD3086 (41.5 days each). The longest vegetative phase was recorded for DBW301 (124 days); this caused it to have the shortest GFD (25.8 days). Higher NDVI values reflected high biomass in the trial. Higher NBI values with lower FLV indicated the nitrogen use efficiency of the genotypes. This was found to be highest in WH1270, followed by PBW825 and DBW187. The high yielding entries DBW303 and DBW187 also showed higher harvest indexes under high fertility conditions.

### 2.3. Correlation Analysis of Traits with Grain Yield

Correlation analysis was performed using Pearson’s correlation coefficient to find the relation of grain yield with different phenological, physiological, and yield attributing traits. Grain yield per plot showed a significant negative correlation (−0.63) with days to heading, while it showed a significant positive correlation with grain filling duration (0.51), biomass (0.73), harvest index (0.75), NDVI (0.27), and TGW (0.62). However, the correlations with other traits were not significant (Table 4).

### 2.4. Partial Budgeting for HYPT

Applications of FYM, growth regulators, and additional fertilizers were calculated based on the prevailing labour cost and the market price of these inputs in 2019. The additional cost was estimated to be USD 203.51 per hectare (Table 5). The benefit to additional grain and straw yield was also calculated. The gain during the HYPT mean yield over the mean of the Advance Varietal Trial (AVT) planted under timely sown irrigated conditions in the same year (2018–2019) [21] was 12.1 q/ha; it was 15.69 q/ha for straw yield. The additional returns from the HYPT were estimated to be USD 405.46 per hectare. Deducting the cost from additional returns, the net benefit of USD 203.51 per hectare was estimated with a B:C ratio of 1.99.

## 3. Discussion

Wheat productivity is highly vulnerable to climate change. The direct impact of climate change leads to wheat yield losses of 1–8% in the Indo-Gangetic Plains [8]. Therefore, adopting convenient mitigation strategies to cope with climate change is necessary for food security. Researchers, particularly plant breeders, are continuously working to develop climate-resilient wheat varieties with an enhanced buffering ability to weather fluctuations, primarily heat and drought. Climate change is predicted to negatively impact wheat yields across northern India, largely due to increased heat stress during grain filling at the end of the growing season. One of the mitigation strategies that farmers may adopt is by sowing wheat earlier in order to avoid terminal heat stress [20,22]. However, often the temperature may not be favourable for early sowing. Therefore, varieties carrying genes for early heat tolerance are necessary for early sowing [20]. The wheat genotypes, except the check varieties, used in this study were bred specifically for early sowing conditions. As such, their testing was expected to identify the best wheat genotypes under high fertility conditions with a suitable agronomic package. This trial, to identify the wheat genotypes best suited for early sowing conditions, was initiated based on a recommendation of a new multilocation trial under the All India Coordinate Wheat and Barley Improvement project in 2018 (https://www.aicrpwheatbarleyicar.in/wp-content/uploads/2021/06/Proceedings.2018.pdf (accessed on 28 November 2022)). The best dose of the fertilizer for early planting was identified during the AICRP experiment; it was reported that the genotypes showed the best performance at 150% of NPK + 15 t/ha FYM [23]. Therefore, the present experiment was carried out using 150% RFD + 15 t/ha FYM, along with two sprays of commercial formulations of 0.2% Chlormequat chloride (CCC) +0.1% Tebuconazole (TBZ) at the first node and flag leaf stage [23].

The mean yield of the SPL-HYPT across six locations was 12.1 q/ha higher than the mean yield of AVT irrigated timely sown trial (62.2 q/ha) of NWPZ in the same season. In addition, the mean yield of the agronomic trial conducted using the same genotypes under RDF in same locations was 68.4 q/ha; this shows that the selected genotypes have higher yield potential when compared to normally sown genotypes [24]. The combined effect of early sowing and the application of growth regulators and higher amounts of fertilizer translated into additional grain yield of 19.4% when compared to the observed 60 q/ha mean yield of wheat in AVT of NWPZ for the past decade [5]. This indicates that the maximum yield potential of superior wheat varieties can be realized by bringing forward sowing dates by 1–2 weeks, providing higher nutrient doses, and preventing losses due to lodging and diseases through the application of CCC and TBZ. These results are in line with many of the following reports, wherein researchers have studied the potential of higher nitrogen application and plant growth regulators in increasing wheat yield potential [25]. To obtain a grain yield of more than 70 q/ha, spring wheat may require more than 300 kg N/ha [26], but achieving high wheat yields in irrigated environments with high N application has been limited by lodging [27]. An abundant nitrogen supply promotes vegetative growth and tillering but produces lanky and succulent culms that are highly susceptible to lodging [28]; this can cause an 8–80% reduction in grain yield and grain quality in wheat [29,30,31,32]. Nitrogen management through the application of a moderate level of N (120 kg/ha) is one of the most common methods to prevent lodging in wheat fields [33,34].

Plant growth regulators (PGRs) that reduce lodging have been evaluated on commercial wheat cultivars under irrigated high fertility conditions. PGRs, including onium compound chlormequat chloride (CCC) and triazole compound tebuconazole (TBZ), have been widely reported as a chemical management strategies to manipulate plant height and reduce lodging in crops [35,36,37]. In its annual progress report of Crop Improvement [21], the AICRP on Wheat and Barley has recommended the use of CCC and TBZ as a tank mix to induce lodging resistance. The application of CCC at the onset of stem elongation reduces the straw length [38]. Tebuconazole is one of the members of the triazole family that acts as a growth regulator as well as a fungicide. Thus, many of these studies have shown the impact of higher nitrogen doses and growth regulators effect on wheat yield in isolation. However, the present study demonstrated the potential of the combined effect of higher nutrient levels of FYM @ 15 t/ha + NPK at 150% RDF +CCC (0.2%) +  TBZ (0.1%) along with early sowing practices to improve wheat yield potential at the field level.

Early heat tolerance with high yield potential is a new subject in wheat breeding [20]. This study revealed that the slight delay in days to heading during early sowing, longer grain filling duration, higher biomass, and higher partitioning of photosynthates to grain in terms of harvest index, canopy greenness, and higher thousand-grain weight, significantly contributed to improving grain yield under early sowing, higher input conditions. Increasing biomass, harvest index, and grain filling duration are proposed as a potential options for crop yield improvement [39,40]. In this study, early sowing appears to have helped in prolonged vegetative growth and an increased grain filling duration to improve the thousand-grain weight of the entries; this is an important factor for improving grain yield. However, other physiological traits, such as CT, CCI, FLV, and photosynthetic efficiency (F_v_/F_m_), which generally reflects the healthiness of the plants, did not vary significantly among the genotypes as they were grown under high input conditions without any stress.

The application of FYM @15 t/ha, NPK at 150% RDF i.e., 225:90:60 kg/ha, and two spays of CCC (0.2%) and TBZ (0.1%) provided an additional net return of USD 201.95 per ha under early sowing conditions of NWPZ with a B:C ratio of 1.99, implying the positive economic benefit of the intervention. Per dollar invested, the adoption of HYPT results in a profit of USD 0.99. It has been reported that sowing agronomically superior wheat varieties with early heat tolerance in the third to fourth week of October can yield up to 80 q/ha [19], and that the application of growth regulators CCC and Tebuconazole provided additional net returns of INR 5862 (~USD 83) [41]. This shows higher yield along with the economic profitability that is necessary to encourage farmers to adopt new technologies.

## 4. Materials and Methods

### 4.1. Sowing Condition and Plant Material

**Figure 2:**
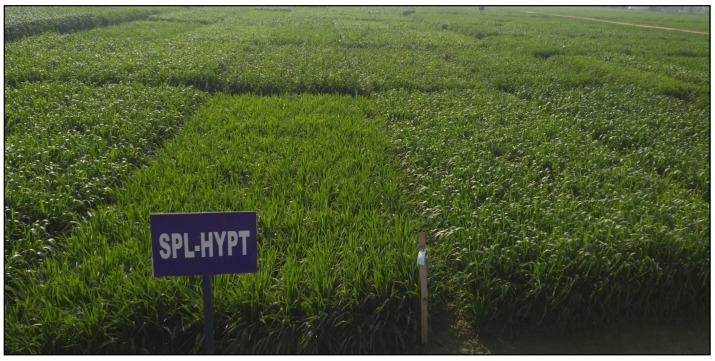
Field view of the SPL-HYPT trial at Karnal, India.

A special wheat yield trial, the ‘High Yield Potential Trial’ (HYPT), was formulated for multilocation testing in the NWPZ during 2018–2019 under the AICRP on Wheat and Barley. Wheat was planted at six locations namely, Gurdaspur, Ludhiana, Ladhowal, Karnal, Delhi, and Pantnagar, spread over three Indian states, Punjab, Haryana, and Uttar Pradesh; these states provide more than half of the wheat to the national buffer stock. This trial consisted of 13 advanced wheat genotypes and two check varities and was planted under early sowing conditions (date of sowing 25 October–5 November) in a randomized block design with four replications (Figure 2). The wheat genotypes belonged to three different institutions and were HD3317, HD3347, DBW187, DBW301, DBW302, DBW303, DBW304, PBW824, PBW825, UP3042, UP3043, WH1254, and WH1270 (Table 6). The checks were HD2967 and HD3086; these are the two leading varieties in Northwest India. These genotypes were contributed by different wheat breeding centres in India, including PAU Ludhiana, Punjab; ICAR-IIWBR Karnal, Haryana; CCSHAU Hisar, Haryana; ICAR-IARI New Delhi; and GBPUA&T Pantnagar, Uttar Pradesh.

### 4.2. Soil and Weather Condition of Study Locations

**Figure 3:**
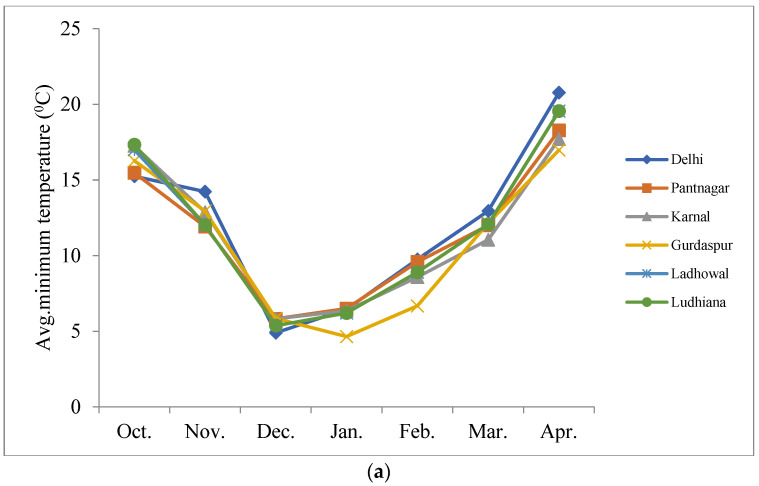

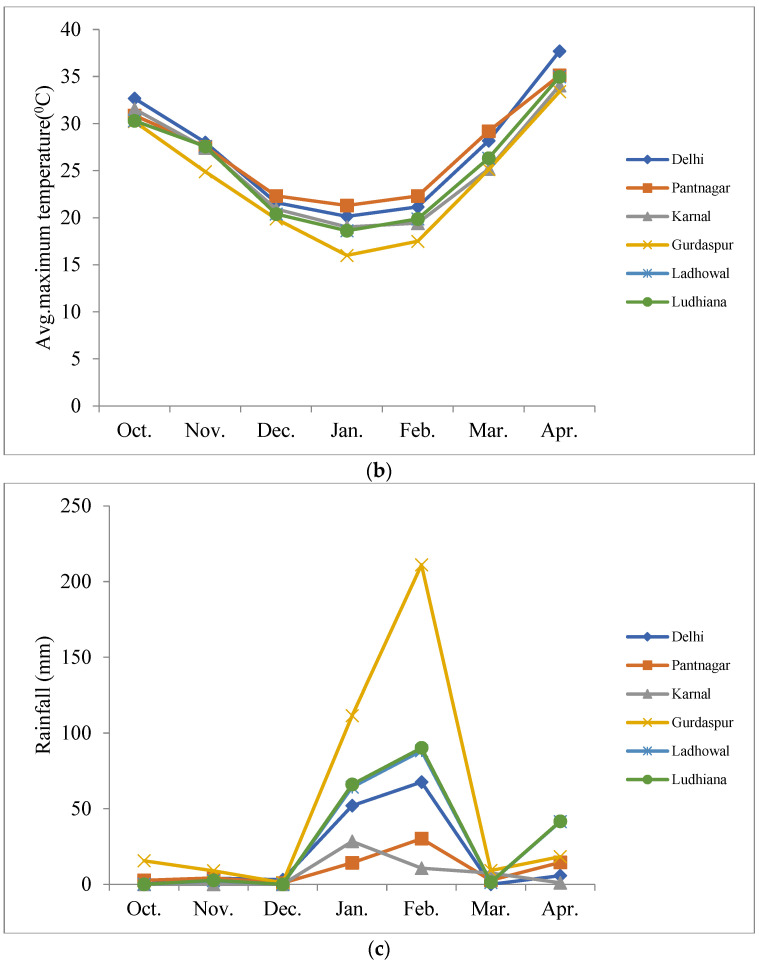
The average minimum (**a**) and maximum (**b**) temperature and rainfall data (**c**) of the study locations during the wheat cropping season.

All the study locations had sandy loam soil except Pantnagar, that had loamy soil. The soil organic carbon varied from 0.37–0.42%, Soil pH from 7.5–8, and EC from 0.25–0.4 dsm^−1^ across the study locations. The avg. minimum temperature (Figure 3a), avg. maximum temperature (Figure 3b), and rainfall (mm) (Figure 3c) recorded for each month during the cropping period in all locations are reported. The temperature trend remains the same across locations and the highest rainfall was recorded at Gurdaspur.

### 4.3. Agronomic Practices

The trial plots, measuring 14.4 m^2^ (12 rows of 6 m, 20 cm apart) were given 150% of the recommended dose of fertilizers: 150 kg of N, 60 kg of P_2_O_5_, and 40 kg of K per ha with 15 tonnes/ha of farmyard manure. These fertilizers and FYM doses were applied based on the recommendation of the AICRP wheat agronomy experiment, that identified maximum yield [23]. Half of the nitrogen was applied as a basal dose in the form of Urea and Diammonium Phosphate (DAP). The remaining nitrogen was applied as a top dressing in two equal split doses at the first (21 days after sowing) and second (45 days after sowing) irrigations. Two sprays of growth regulators, Chlormequat chloride (CCC) @ 0.2% + Tebuconazole (Folicur 250 EC) @ 0.1% of commercial product dose as a tank mix, were applied at the first node (Zadoks scale31) and flag leaf (Zadoks scale 39) stage [42]. Based on the soil moisture level, four to six irrigations were provided to the trials. The trials were kept free from weeds through a combination of hand weeding and chemical control. Two sprays of Tebuconazole served the dual role of growth regulation and control of diseases, particularly yellow and brown rusts. All twelve rows of the plots were harvested for recording yield and biomass. The grain yield per plot (GYPP) in kg was recorded at all six locations and then converted to grain yield in quintal/hectare (q/ha).

### 4.4. Yield, Phenological, and Physiological Traits Measurement

Data recording at different crop growth stages was carried out using the standard Zadoks reference scale [42]. The yield attributing traits, like plant height (PH), tiller number/m (TN), biomass (BM), spike length (SPKL) in cm, grain numbers per spike (GNS), grain weight per spike (GWS), thousand-grain weight (TGW), and harvest Index (HI), were measured. Phenological traits, including days to heading (DTH), days to maturity (DTM), and grain filling duration (GFD), which was calculated from the days to anthesis to days to physiological maturity, were recorded. Physiological traits such as chlorophyll fluorescence (Fv/Fm) (Model OS30P+, Opti-Sciences, Inc., Hudson, NH, USA), which measures the photosystem II efficiency and, indirectly, the photosynthetic efficiency of the genotype, nitrogen balance index (NBI)(Force, DUALEX^R^, Ocala, FL, USA), which indicates the nitrogen use efficiency of the genotype, flavonol content index (FLAV) (Force, DUALEX^R^, Ocala, FL, USA), and canopy temperature (CT)(HTC MT-4), which indicates the stress tolerance of the genotypes, chlorophyll content index (CCI) (Chlorophyll meter, SPAD-502 Plus, Konica Minolta, Chiyoda-ku, Tokyo, Japan.), and normalized difference vegetation Index (NDVI)(Trimble industries, Inc., Westminster, CO, USA), which measures the greenness of the genotypes, were recorded. These physiological traits, that significantly indicate the healthiness of the plant which, inturn, contributes to the improved yield potential in the genotypes, were recorded at maximum vegetative stage (Zadok’s scale 41) using the specific instruments (as mentioned in the parenthesis) at ICAR-IIWBR, Karnal [43].

### 4.5. Statistical Analysis

The recorded traits were analyzed to determine their contribution to the increase in grain yield during the HYPT trial using SPSS version 21 [44]. The analysis of the variance model for RBD was:

Y_ij_ = µ + t_i_ + r_j_ + e_ij_

where,

µ is the overall mean;

t_i_ is the ith treatment effect;

r_j_ is the jth replication effect; and

e_ij_ is the error term.

In the analysis of variance and the variance estimates, a mixed model approach was followed, and the model is shown below.

Y = checks + location + checks × location + genotypes + genotypes × location + error

The mean grain yield of a genotype was also expressed as the percentage of the best check using the following formula:

% GY = (GYg/GYc) × 100

GYg is the mean grain yield of a genotype and GYc is the mean grain yield of the local check.

The traits were analyzed using critical difference (CD) and Duncan tests (GenStat 18th Edition, VSN International Ltd., Hemel Hempstead, UK) to find the significant differences of the traits among study genotypes. The correlation of different traits to yield was assessed using Pearson’s correlation (SAS 9.3 Inc., Cary, NC, USA).

### 4.6. Partial Budgeting of HYPT Trial

A popular planning and decision-making tool was employed to assess the impact of the HYPT intervention using the information on costs and returns incurred from the intervention. In this study, the focus will only be on the change brought about by the intervention without estimating the complete budget. It explicitly indicates the level of profitability from the intervention (HYPT) by computing the net gain from the suggested change or refinement in the package of practices. This is conducted by estimating the difference between ‘total benefits’ and ‘total costs’ owing to the HYPT. Two items are considered under ‘total benefits’: ‘what are the added revenues?’, and what are the reduced costs? Likewise, under ‘total costs’, the two items considered were: ‘what are the additional costs?’ and ‘what are the reduced revenues?’. A positive net gain value indicates the economic viability of the intervention and vice-versa, and the highest value is chosen when compared between two or more interventions [45,46].

## 5. Conclusions

Improving climate resilience in the wheat improvement program is necessary to sustain production and productivity. This study facilitates a technology using a combination of high yielding genotypes, early sowing, and the application of FYM @15 t/ha, NPK at 150% RDF along with two sprays of CCC (0.2%) and Tebuconazole (0.1%) to provide increased returns to farmers by addressing both heat stress tolerance and higher economic return. As a result of this study, widely adopted high yielding wheat varieties, including DBW187, DBW303, and WH1270, were released to farmers for cultivation. In addition, by looking into its monetary benefits, the HYPT was adopted as one of the special trials in the AICRP wheat program of India to identify and release high yield wheat varieties adapted to early sowing for the benefit of the farmers and to sustain wheat production in the country to ensure food security. The results obtained prove that this kind of need-based research is crucial for improving wheat productivity to cope with climate change effects that have been forecasted to challenge food security for poor and smallholder farmers.

## Figures and Tables

**Table 1:** Mean yield (q/ha) of HYPT trials at six locations during 2018–2019.

SN	Genotype	Gurdaspur	Ludhiana	Ladhowal	Karnal	Delhi	Pantnagar	Zonal Mean	% Gain over Best Check	Zonal Mean under RDF
1	HD3317	63.4	82.1	63.3	84.5	73.0	74.0	73.7	−2.51	65.8
2	WH1254	60.1	75.4	69.9	80.4	66.0	72.4	70.7	−6.48	72.2
3	DBW301	66.7	79.1	68.4	78.0	71.4	66.6	71.7	−5.16	62.3
4	WH1270	78.3	84.6	67.5	91.5	77.2	71.8	78.5	3.84	71.4
5	PBW824	66.5	87.2	68.0	98.8	68.4	71.1	76.7	1.46	69.7
6	UP3043	67.2	74.8	70.1	92.9	77.0	77.7	76.6	1.32	71.3
7	DBW187	75.6	77.4	74.6	96.6	70.9	76.5	78.6	3.97	73.0
8	DBW303	73.0	91.9	70.9	97.4	69.7	79.2	80.4	6.35	71.3
9	DBW304	66.3	77.4	49.5	87.8	62.2	71.0	69.0	−8.73	64.3
10	UP3042	68.3	86.7	64.7	96.4	73.2	72.3	76.9	1.72	69.1
11	DBW302	60.1	79.2	69.4	89.2	70.4	79.3	74.6	−1.32	66.7
12	PBW825	69.1	82.4	67.7	86.2	75.1	68.4	74.8	−1.1	65.6
13	HD3347	62.6	81.9	63.5	85.2	67.0	67.3	71.2	−5.82	67.6
14	HD2967(C)	38.1	78.4	67.8	81.6	59.5	72.0	65.9	−12.83	64.7
15	HD3086(C)	69.9	80.5	70.4	88.3	70.3	73.9	75.6	−	70.5
Grand Mean		65.7	81.3	67.2	89.0	70.0	72.9	74.3	−	68.4
AVT-IR-TS		−	−	−	−	−	−	62.2		−
SE		1.94	3.23	2.09	1.91	3.11	0.47	0.95	−	−
CD		4.6	7.7	5.0	4.6	7.4	1.1	2.2	−	−
CV		5.9	8.0	6.2	4.3	8.9	1.3		−	−

AVT-IR-TS—Advanced varietal trial-Irrigated-Timely sown, SE—Standard error, CD—Critical difference, CV—Coefficient of variation, RDF—Recommended dose of fertilizer.

**Table 2:** Various phenological and physiological traits recorded in HYPT.

Genotypes	DTH (Days)	DTM (Days)	GFD (Days)	Fv/Fm	NBI	FLV	CCI	CT (°C)	NDVI
HD3717	115 ± 2.6 ^b^	156 ± 2.7 ^a^	42 ± 0.28 ^b^	0.787 ± 0.012 ^abc^	33.08 ± 4.01 ^b^	1.258 ± 0.10 ^ab^	39.6 ± 1.8 ^a^	26.6 ± 1.3 ^a^	0.905 ± 0.003 ^abc^
WH1254	111± 1.9 ^b^	152 ± 1.7 ^c^	42 ± 0.28 ^b^	0.767 ± 0.014 ^bc^	30.05 ± 4.64 ^ab^	1.218 ± 0.07 ^ab^	35.4 ± 6.6 ^a^	26.3 ± 1.3 ^a^	0.895 ± 0.009 ^abc^
DBW301	119 ± 5.7 ^a^	148± 1.7 ^d^	29 ± 3.94 ^f^	0.810± 0.004 ^ab^	25.27 ± 0.16 ^ab^	1.355 ± 0.05 ^ab^	33.9 ± 2.0 ^a^	27.9 ± 1.2 ^a^	0.910 ± 0.004 ^a^
WH1270	108 ± 2.6 ^fg^	147 ± 4.0 ^gh^	39 ± 1.43 ^d^	0.812 ± 0.006 ^ab^	41.20 ± 1.58 ^a^	1.013 ± 0.05 ^b^	41 ± 1.0 ^a^	27.2 ± 1.7 ^a^	0.887 ± 0.006 ^cdef^
HD2967 (C)	115 ± 3.1 ^be^	154 ± 1.7 ^b^	40 ± 1.65 ^cd^	0.777 ± 0.011 ^bc^	35.28 ± 7.34 ^ab^	1.390 ± 0.15 ^ab^	32.0 ± 5.2 ^a^	27.5 ± 1.7 ^a^	0.900 ± 0.004 ^abcd^
PBW824	109 ± 4.7 ^h^	149 ± 0.2 ^de^	40± 4.42 ^a^	0.786 ± 0.010 ^abc^	27.58 ± 2.39 ^ab^	1.265 ± 0.06 ^ab^	34.7 ± 1.9 ^a^	27.6 ± 1.0 ^a^	0.890 ± 0.004 ^bcde^
UP3043	105± 0.3 ^gh^	145 ± 1.0 ^f^	41 ± 0.95 ^b^	0.789 ± 0.005 ^abc^	31.65 ± 4.51 ^ab^	1.585 ± 0.12 ^a^	36.7 ± 3.3 ^a^	27.1 ± 1.0 ^a^	0.883 ± 0.006 ^ef^
DBW187	108 ± 0.8 ^ef^	148 ± 0.2 ^e^	41 ± 0.62 ^b^	0.782 ± 0.011 ^abc^	36.62 ± 2.45 ^ab^	1.058 ± 0.05 ^b^	38.0 ± 1.2 ^a^	27.8 ± 1.0 ^a^	0.882 ± 0.005 ^def^
HD3086 (C)	105 ± 4.4 ^j^	145 ± 3.3 ^h^	40 ± 1.10 ^b^	0.759 ± 0.012 ^c^	29.17 ± 1.59 ^ab^	1.160 ± 0.09 ^ab^	32.0 ± 0.9 ^a^	27.9 ± 1.7 ^a^	0.893 ± 0.007 ^bcdef^
DBW303	103 ± 1.0 ^i^	143 ± 0.5 ^g^	41 ± 1.25 ^b^	0.779 ± 0.005 ^abc^	32.85 ± 2.54 ^ab^	1.185 ± 0.04 ^ab^	38 ± 2.1 ^a^	27.1 ± 2.3 ^a^	0.885 ± 0.003 ^def^
DBW304	109 ± 4.4 ^h^	148 ± 3.7 ^g^	39 ± 0.70 ^bc^	0.795 ± 0.009 ^abc^	30.52 ± 1.27 ^ab^	1.183 ± 0.02 ^b^	35.6 ± 2.4 ^a^	28.8 ± 2.3 ^a^	0.890 ± 0.004 ^bcdef^
UP3042	109 ± 0.6 ^d^	148 ± 0.4 ^e^	39 ± 0.94 ^cd^	0.771 ± 0.014 ^bc^	28.37 ± 4.73 ^ab^	1.228 ± 0.09 ^ab^	32.6 ± 3.9 ^a^	26.3 ± 0.3 ^a^	0.908 ± 0.002 ^ab^
DBW302	121 ± 3.0 ^a^	157 ± 2.2 ^a^	36± 0.85 ^e^	0.819 ± 0.005 ^a^	27.81 ± 0.41 ^ab^	1.315 ± 0.03 ^ab^	36.5 ± 0.7 ^a^	26.7 ± 0.5 ^a^	0.908 ± 0.006 ^ab^
PBW825	112 ± 0.5 ^b^	151 ± 1.2 ^d^	39 ± 1.03 ^cd^	0.768 ± 0.003 ^bc^	36.75 ± 2.78 ^ab^	1.098 ± 0.10 ^b^	39.5 ± 2.1 ^a^	26.6 ± 1.5 ^a^	0.883 ± 0.009 ^f^
HD3347	106 ± 1.9	147 ± 1.5 ^e^	41 ± 0.47 ^b^	0.794 ± 0.002 ^abc^	29.28 ± 0.63 ^ab^	1.213 ± 0.03 ^ab^	35.2 ± 1.8 ^a^	27.8 ± 0.7 ^a^	0.900 ± 0.007 ^abc^
CD	8.922	6.320	5.265	0.025	N/A	0.247	N/A	N/A	0.016
SE	3.115	2.207	1.838	0.009	3.102	0.086	3.02	1.38	0.006
CV	5.650	2.950	9.390	2.240	19.57	13.950	4.26	1.95	1.25

Means followed by different letters in each column are not significantly different at *p* = 0.05, Duncan Multiple Range Test.

**Table 3:** Various yield associated traits recorded during HYPT.

Genotypes	BM (kg)	HI	GNS	GWS (g)	TGW (g)	PH (cm)	SPKL (cm)	TN
HD3717	32.7 ± 0.6 ^ab^	38.5 ± 1.21 ^cd^	51 ± 0.5 ^e^	2.5 ± 0.0 ^d^	48.9 ± 0.4 ^def^	104.0 ± 2.273 ^ab^	10.2 ± 0.49 ^cd^	113 ± 2.9 ^abc^
WH1254	31.2 ± 1.5 ^b^	38.5 ± 0.46 ^bc^	42 ± 0.3 ^i^	2.4 ± 0 ^i^	40 ± 0.5 ^i^	100.2 ± 1.601 ^cdef^	11.8 ± 0.71 ^abcd^	113 ± 9.1 ^abc^
DBW301	32.6 ± 1.0 ^ab^	35.9 ± 2.19 ^d^	53 ± 0.2 ^cd^	2.0 ± 0.0 ^h^	40.3 ± 0.3 ^i^	98.5 ± 2.327 ^f^	13.57 ± 0.17 ^a^	105 ± 11.8 ^abc^
WH1270	33.9 ± 1.7 ^ab^	37.8 ± 1.67 ^bc^	48 ± 0.3 ^f^	2.4 ± 0.0 ^de^	50.1 ± 0.4 ^cd^	95.7 ± 1.315 ^ef^	11.40 ± 0.44 ^abcd^	114 ± 8.7 ^abc^
HD2967 (C)	32.5 ± 0.7 ^ab^	37.9 ± 1.08 ^cd^	45 ± 0.6 ^h^	2.0 ± 0.0 ^h^	45.1 ± 0.4 ^h^	104.2 ± 2.056 ^ab^	11.22 ± 0.66 ^bcd^	102 ± 13.4 ^abc^
PBW824	32.8 ± 2.1 ^a^	41.9 ± 1.20 ^abc^	45 ± 0.3 ^gh^	2.1 ± 0.0 ^gh^	45.5 ± 0.2 ^h^	100.8 ± 1.887 ^bce^	11.17 ± 0.08 ^bcd^	101 ± 4.9 ^abc^
UP3043	34 ± 1.1 ^ab^	39.3 ± 1.68 ^abc^	53 ± 0.5 ^c^	2.8 ± 0.03 ^bc^	53.0 ± 0.5 ^b^	108.0 ± 2.121 ^ab^	10.80 ± 0.28 ^bcd^	89 ± 7.7 ^c^
DBW187	34.2 ± 1.7 ^ab^	41.5 ± 1.30 ^ab^	47 ± 0.5 ^fg^	2.9 ± 0 ^b^	62 ± 0.5 ^a^	104.0 ± 2.160 ^bcd^	11.05 ± 0.73 ^bcd^	126 ± 18.3 ^ab^
HD3086 (C)	30.7 ± 1.2 ^b^	40.7 ± 1.89 ^ab^	42 ± 0.6 ^j^	2.4 ± 0 ^j^	46.8 ± 0.2 ^g^	98.8 ± 1.109 ^cdef^	10.82 ± 0.51 ^bcd^	133 ± 3.1 ^a^
DBW303	32.5 ± 0.9 ^ab^	42.8 ± 0.70 ^a^	65 ± 0.9 ^a^	3.2 ± 0.0 ^a^	48.2 ± 0.4 ^ef^	98.5 ± 1.041 ^def^	10.95 ± 0.67 ^bcd^	103 ± 6.2 ^abc^
DBW304	32.3 ± 1.3 ^ab^	39.5 ± 0.73 ^abc^	51 ± 0.4 ^e^	2.5 ± 0.0 ^d^	49.2 ± 0.6 ^cde^	101.0 ± 2.041 ^cdef^	12.57 ± 0.32 ^abc^	109 ± 11.8 ^abc^
UP3042	33.5 ± 1.2 ^ab^	39.5 ± 1.34 ^abc^	47 ± 0.2 ^fg^	2.3 ± 0.03 ^ef^	50.4 ± 0.3 ^c^	105.7 ± 2.097 ^abc^	9.72 ± 0.37 ^d^	96 ± 2.1 ^abc^
DBW302	32.9 ± 1.3 ^ab^	38.9 ± 1.79 ^cd^	58 ± 0.2 ^b^	2.8 ± 0.0 ^b^	48.5 ± 0.2 ^ef^	109.5 ± 1.936 ^ab^	11.00 ± 0.28 ^bcd^	129 ± 3.7 ^ab^
PBW825	30.6 ± 0.5 ^b^	39.4 ± 1.44 ^abc^	52 ± 0.6 ^de^	2.7 ± 0.0 ^c^	50.2 ± 0.4 ^cd^	101.7 ± 2.175 ^bcd^	11.55 ± 0.60 ^abcd^	103 ± 12.8 ^abc^
HD3347	30.7 ± 0.7 ^b^	40.3 ± 0.75 ^abc^	44 ± 0.3 ^h^	2.2 ± 0.0 ^fg^	47.8 ± 0.3 ^fg^	98.7 ± 1.031 ^def^	12.67 ± 0.40 ^ab^	104 ± 11.8 ^bc^
CD	N/A	N/A	1.437	0.146	1.154	5.315	1.399	N/A
SE	1.16	1.369	0.49	0.05	0.396	1.856	0.488	9.36
CV	7.17	6.92	0.70	0.071	0.56	3.64	8.58	17.09

DTH—days to heading, DTM—days to maturity, GFD—grain filling duration, Fv/Fm—variable fluorescence/maximal fluorescence, NBI—nitrogen balance index, FLV—flavanoid index, CCI—chlorophyll content index, CT—canopy temperature, NDVI—normalized difference vegetation index, BM—Biomass (kg/plot), HI—harvest Index, GNS—grain number/spike, GWS—grain weight/spike, TGW—thousand grain weight, PH—plant height, SPKL—spike length, TN—tiller number/meter, CD—critical difference, SE—standard error, CV—coefficient of variation. Means followed by different letters in each column are not significantly different at *p* = 0.05, Duncan Multiple Range Test

**Table 4:** Correlation coefficients of traits with grain yield.

Traits	r	*p* Values
PH	0.24	0.393
TN	−0.33	0.23
DTH	**−0.63**	0.013 *
DTM	−0.45	0.096
GFD	**0.51**	0.04 *
NDVI	**0.27**	0.047 *
BM	**0.73**	0.002 **
HI	**0.75**	0.001 **
F_V_/F_m_	−0.05	0.862
NBI	0.22	0.435
CCI	0.32	0.251
FLAV	−0.38	0.164
CT	0.27	0.326
SPKL	−0.20	0.473
GNS	0.23	0.405
GWS	0.48	0.068
TGW	**0.62**	0.014 *

* *p* < 0.05, ** *p* < 0.01.PH—Plant height, TN—tiller number/meter, DTH—days to heading, DTM—days to maturity, GFD—grain filling duration, NDVI—normalized difference vegetation index, BM—biomass, HI—harvest Index, Fv/Fm—variable fluorescence/maximal fluorescence, NBI—nitrogen balance index, CCI—chlorophyll content index, FLV—flavanoid index, CT—canopy temperature, SPKL—spike length, GNS—grain number/spike, GWS—grain weight/spike, TGW—thousand grain weight.

**Table 5:** Partial budgeting estimates for HYPT.

Additional Cost (Annualized)	USD	Additional Returns (Annualized)	USD
1. 150% RFD (Nutrients Cost)	31.90	1. Grain (+12.1 Q/ha)	316.29
2. Lihocin: 2 L/ha	25.57	2. Straw (+15.69 Q/ha)	89.16
3. Folicur 430: 1 L/ha	31.25	--	--
4. FYM Cost: 15 t/ha	68.19	--	--
5. Labour Cost for all inputs	46.60	--	--
**Reduced Returns (annualized)**	**USD**	**Reduced Cost (annualized)**	**USD**
Nil	--	Nil	--
Total Negative Effects	203.51	Total Positive Effects	405.46
Net Gain			201.95

Exchange Rate (2019): 1 USD = INR 70.39.

**Table 6:** Pedigree details of the genotypes used in the study.

SN	Genotype	Pedigree
1.	HD3317	31stESWYT-117//DW1272/HP1731
2.	WH1254	PRL/2*PASTOR//PBW343*2/KUKUNA/WHEAR//INQALAB91*2/TUKURU//SOKOLL*2/4/CHEN/AEGILOPS SQUARROSA(TAUS.)
3.	DBW301	SR39/DPW621-50
4.	WH1270	SHA7//PRL/VEE#6/3/FASAN/4/HAAS8446/2*FASAN/5/CBRD/KAUZ/6/MILAN/AMSEL/7/FRET2*2/KUKUNA/8/2*WHEAR/SOKOLL
5.	PBW824	WAXWING//INQALAB91*2/KUKUNA/3/WBLL1*2/TUKURU/8/2*NG8201/KAUZ/4/SHA7//PRL/VEE#6/3/FASAN/5/MILAN/KAUZ/6/ACHYUTA/7/PBW343*2/KUKUNA
6.	UP3043	CHIBIA//PRLII/CM65531/3/SKAUZ/BAV92*2/4/HUW234+LR34/PRINIA//PBW343*2/KUKUNA/3/ROLF07
7.	DBW187	NAC/TH.AC//3*PVN/3/MIRLO/BUC/4/2*PASTOR/5/KACHU/6/KACHU
8.	DBW303	WBLL1*2/BRAMBLING/4/BABAX/LR42//BABAX*2/3/SHAMA*2/5/PBW343*2/KUKUNA*2//FRTL/PIFED
9.	DBW304	ADI/3/KINGBIRD#1//INQALAB91*2/TUKURU/4/NADI
10.	UP3042	CAL/NH//H567.71/3/SERI/4/CAL/NH//H567.71/5/2*KAUZ/6/WH576/7/WH542/8/WAXWING/9/ATTILA*2/PBW65/6/PVN//CAR422/ANA/5/BOW/CROW//BUC/PVN/3/YR/4/TRAP#1/7/ATTILA/2*PASTOR/10/UP2338*2/KKTS*2//YA/NAC
11.	DBW302	DBW112/HD3108
12.	PBW825	SAUAL/MUTUS*2//PICAFLOR #1
13.	HD3347	HD3086/HD2997
14.	HD2967	ALD/CUC//URES/HD2160M/HD2278
15.	HD3086	DBW14/HD2733//HUW468

## Data Availability

Not applicable.
